# Echocardiographic mechanical dyssynchrony predicts long-term mortality in patients with cardiac resynchronisation therapy

**DOI:** 10.1007/s10554-023-02972-1

**Published:** 2023-10-11

**Authors:** Mohamed Abdelbaset Ahmed, Muhammed Gercek, Philipp Sommer, Volker Rudolph, Daniel Dumitrescu, Lothar Faber, Henrik Fox

**Affiliations:** 1 Clinic for General and Interventional Cardiology/Angiology, Herz- und Diabeteszentrum NRW, Ruhr-Universität Bochum, Georgstr. 11, D-32545 Bad Oeynhausen, Germany; 2 Clinic for Electrophysiology, Herz- und Diabeteszentrum NRW, Ruhr-Universität Bochum, Georgstr. 11, D-32545 Bad Oeynhausen, Germany; 3 Clinic for Thoracic and Cardiovascular Surgery, Herz- und Diabeteszentrum NRW, Ruhr-Universität Bochum, Georgstr. 11, D-32545 Bad Oeynhausen, Germany; 4 Heart Failure Department, Herz- und Diabeteszentrum NRW, Ruhr-Universität Bochum, Georgstr. 11, D-32545 Bad Oeynhausen, Germany

**Keywords:** Apical rocking, Septal flash, Mechanical dyssynchrony, Long-term survival, Cardiac resynchronisation therapy

## Abstract

Cardiac resynchronisation therapy (CRT) is an established treatment for patients with symptomatic heart failure with reduced left ventricular ejection fraction (LVEF ≤ 35%; HFrEF) and conduction disturbances (QRS duration ≥ 130 ms). The presence of mechanical dyssynchrony (MD) on echocardiography has been hypothesised to be of predictive value in determining indication for CRT. This study investigated the impact of MD (apical rocking [AR] and septal flash [SF]) on long-term survival in CRT recipients. HFrEF patients (n = 425; mean age 63.0 ± 10.6 years, 72.3% male, 60.7% non-ischaemic aetiology) with a guideline-derived indication for CRT underwent device implantation. MD markers were determined at baseline and after a mean follow-up of 11.5 ± 8.0 months; long-term survival was also determined. AR and/or SF were present in 307 (72.2%) participants at baseline. During post-CRT follow-up, AR and/or SF disappeared in 256 (83.4%) patients. Overall mean survival was 95.9 ± 52.9 months, longer in women than in men (109.1 ± 52.4 vs. 90.9 ± 52.4 months; p < 0.001) and in younger (< 60 years) versus older patients (110.6 ± 53.7 vs. 88.6 ± 51.1 months; p < 0.001). Patients with versus without MD markers at baseline generally survived for longer (106.2 ± 52.0 vs. 68.9 ± 45.4 months; p < 0.001), and survival was best in patients with resolved versus persisting MD (111.6 ± 51.2 vs. 79.7 ± 47.6 months p < 0.001). Age and MD at baseline were strong predictors of long-term survival in HFrEF patients undergoing CRT on multivariate analysis. Novel echocardiography MD parameters in HFrEF CRT recipients predicted long-term mediated better outcome, and survival improved further when AR and/or SF disappear after CRT implantation.

**Graphical abstract:**

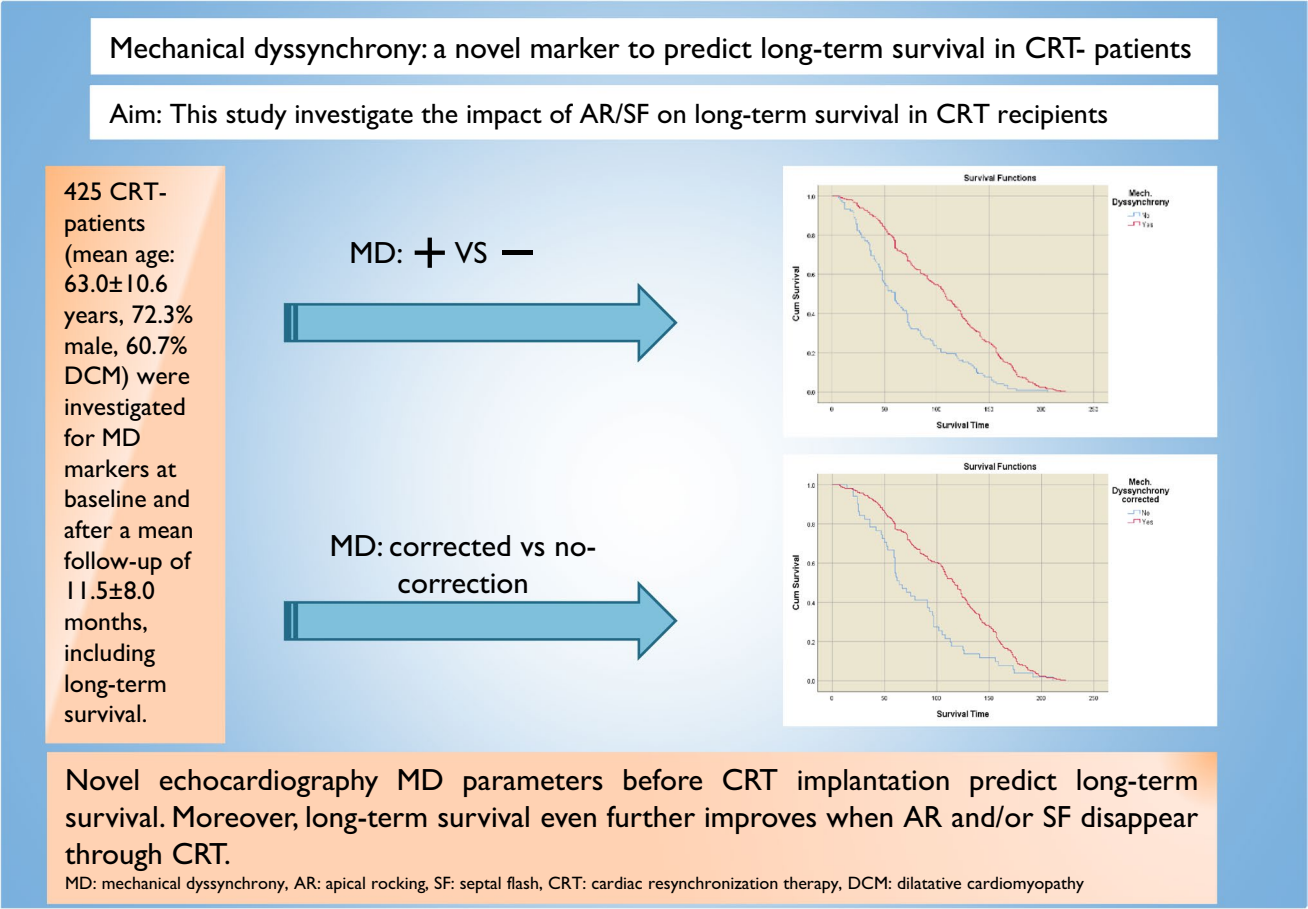

## Introduction

Cardiac resynchronisation therapy (CRT) represents an established treatment modality in patients with symptomatic heart failure with reduced left ventricular ejection fraction (LVEF ≤ 35%; HFrEF) who have conduction disturbances indicated by prolonged QRS duration (≥ 130 ms). Controversially, more than one-third of patients treated with CRT do not benefit from therapy, and reasons for non-response to CRT are the subject of ongoing discussion [1, 2].

**Fig. 1:**
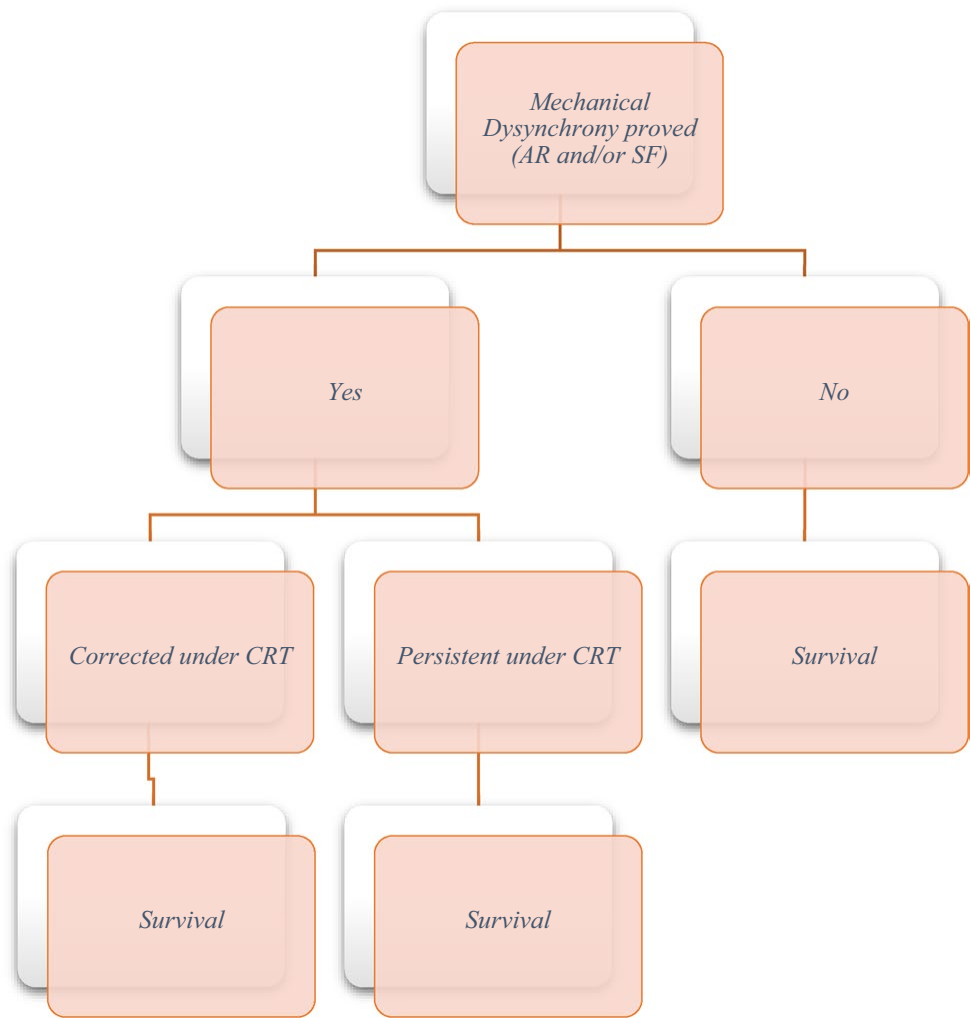
Structure and course of the study. *AR* apical rocking, *CRT* cardiac resynchronisation therapy, *SF* septal flash

**Fig. 2:**
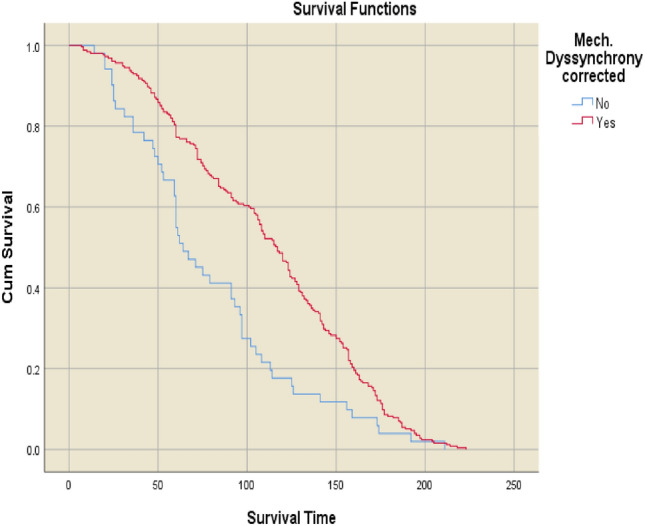
Kaplan–Meier survival curves for patients with versus without mechanical dyssynchrony on baseline echocardiography

**Fig. 3:**
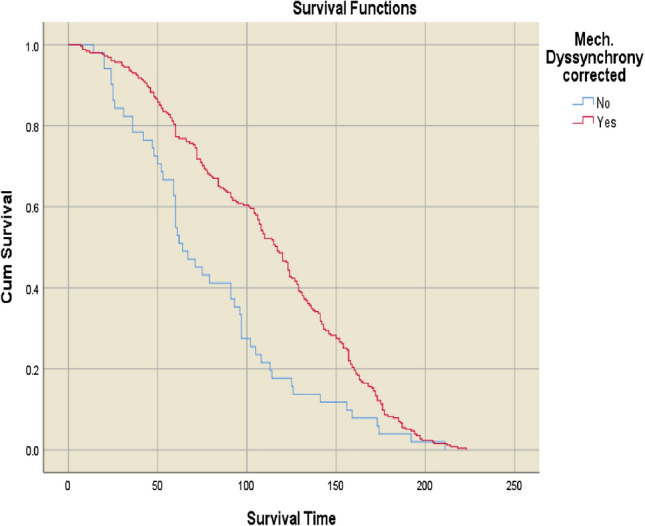
Kaplan–Meier survival curves for patients with versus without resolution of mechanical dyssynchrony after cardiac resynchronisation therapy

Various attempts have been made to better understand this topic, including echocardiography analysis of parameters such as mechanical dyssynchrony, to allow more precise prediction of who will respond to CRT and who will not [3]. However, measures of echocardiographic dyssynchrony have not yet been studied in detail in this context and are still not referred to in current guidelines. This is because data remain inconsistent and due to the large number of other parameters discussed [4, 5] (Figs. 1, 2 and 3).

**Fig. 4:**
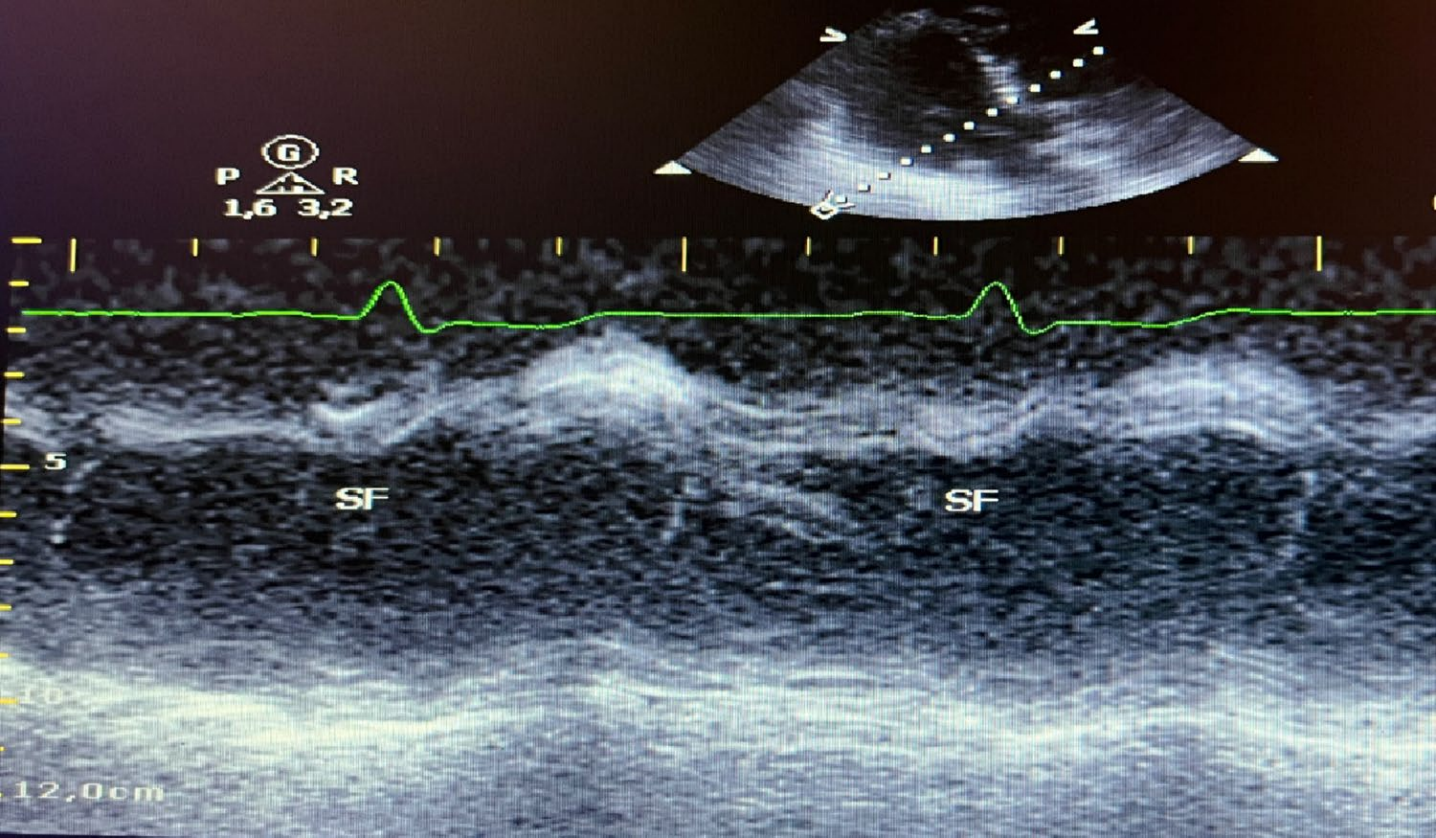
Example of Septal flash (SF) simply identified on 2D echocardiography using M-Mode, SF represented as a short inward early systolic motion of the septum due to the early septal excitation and contraction

**Fig. 5:**
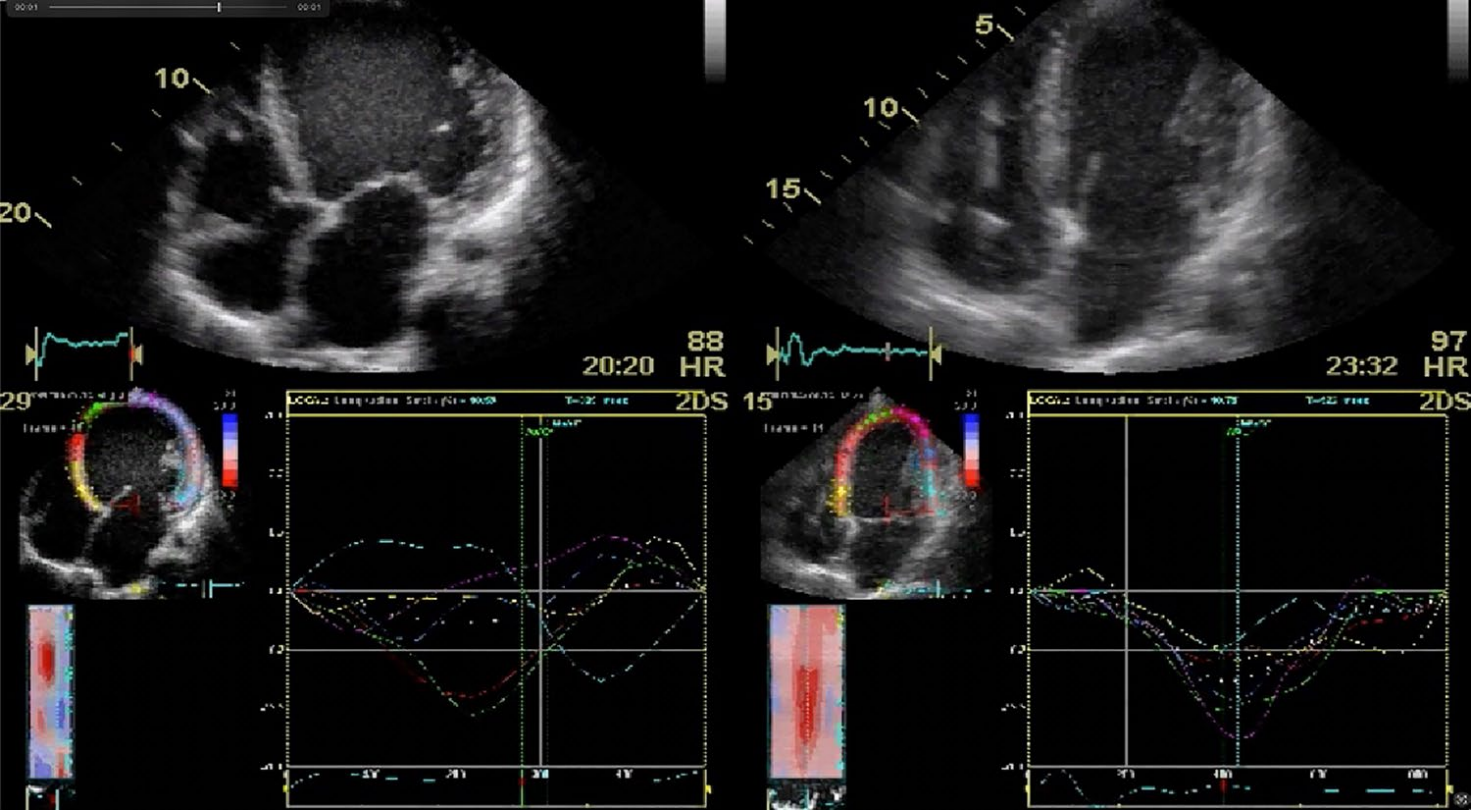
Example of Apical rocking in 2D images, apical rocking occurs due to early activation and contraction of the septum pulling the apex towards right ventricle (marked below in yellow and red) followed by delayed activation and contraction of the lateral wall pulling the apex back to the left (marked below in blue and turquoise) and stretching the relaxed septum

Septal flash (SF) and apical rocking (AR) are two promising echocardiographic signs of mechanical dyssynchrony. SF represents the fast, early systolic and inward septal movement that occurs as a result of early excitation of the septal myocardium by the still-preserved conduction proximal to a bundle branch block [6, 7]. In principle, SF was initially observed by Feigenbaum in patients with left bundle branch block (LBBB) in 1974 [8], and first described as a dyssynchrony parameter in CRT patients by Parsai in 2008 [9]. Echocardiographically, SF can be qualitatively identified or quantified using 2D-mode (“eyeballing”), M-mode or using speckle tracking technology (Figs. 4 and 5) [10].

AR refers to a rocking movement of the left ventricular apical wall that occurs during systole. This rocking motion arises from a brief early systolic forward septal movement, followed by displacement of the apex by shortening of the late-excited posterolateral wall [11]. AR can also be easily visually evaluated using 2D-mode or by measuring the apical transverse motion (ATM) in echocardiography [12].

Both parameters are therefore based on the same pathophysiology and are direct consequences of the mechanical dyssynchrony induced by LBBB [13]. As signs of mechanical dyssynchrony, both AR and SF as mechanical dyssynchrony signs have been shown to have predictive value in short- and medium-term follow-up after CRT [13], but no trial has yet evaluated the long-term prognostic value of these parameters. Therefore, this study investigated the long-term prognostic value of AR and SF as signs of mechanical dyssynchrony in patients with HFrEF undergoing CRT.

## Methods

### Study design and population

This study included patients with HFrEF who had a CRT system implanted for advanced chronic symptomatic heart failure at our centre between 1999 and 2010. Individual decision on CRT implantation was based on the European Society of Cardiology guidelines at the time (LVEF ≤ 35%, QRS duration ≥ 120 ms, New York Heart Association [NYHA] class II-IV and optimal medical therapy for at least 3 months). Heart failure aetiology was either ischaemic or non-ischaemic; patients with ischaemic heart failure were checked for revascularisation options before CRT implantation. The study was approved by institutional ethics committee. Study participants were divided in two groups based on the presence or absence of AR and/or SF (mechanical dyssynchrony) at baseline (Fig. 1). Those with mechanical dyssynchrony were further divided into two subgroups depending on whether mechanical dyssynchrony persisted or disappeared during follow-up after CRT implantation (Fig. 1).

### Assessments

All enrolled patients underwent echocardiography at baseline (prior to CRT implantation) and at 12 months after CRT implantation. Documentation of AR and/or SF at baseline was defined as mechanical dyssynchrony. The resolution of mechanical dyssynchrony after CRT was referred to as mechanical dyssynchrony correction (MDC). Data on patient survival was collected each month over a 10-year period.

### Outcomes

In addition to mechanical dyssynchrony, other endpoints included long-term survival (including implantation of additional cardiac devices or mechanical circulatory support [left ventricular assist device; LVAD], heart transplantation, and explantation of the CRT system [e.g. due to infection]).

### Statistical analysis

All data were prospectively collected and analysed. Electronic patient file and electronic hospital data system records were used to obtain the baseline characteristics of study patients, and relevant parameters during follow up. Private practice cardiologists and primary care providers were also contacted to provide information about long-term patient outcomes and endpoint-related events in enrolled patients.

All study data were captured electronically and statistical analysis of the validated data after database clearance was conducted using commercial software SPSS Version 20 (IBM Corporation, Armonk, New York, USA). Continuous variables are reported as mean ± standard deviation. After checking for normal distribution, variables were examined using the Student’s t test. Non-parametric variables were analysed using the Wilcoxon signed rank test. Categorical variables are reported as whole numbers with percentages. The Chi-square test was used for between-group comparisons. A p value of ≤ 0.05 was defined as the cut-off for statistical significance.

The Kaplan–Meier method was used for long-term survival analysis, also using a significance level of p ≤ 0.05. Multiple regression analysis was used to compare all available variables to identify predictors of survival in post-CRT patients.

## Results

### Study population

A total of 425 HFrEF patients were included (mean age 63.0 ± 10.6 years, 73% male) (Table 1). The majority of patients (60.7%) had non-ischaemic heart failure aetiology, left ventricular function was poor, and almost all were being treated with a β-blocker and a renin-angiotensin-aldosterone system blocker (often with the addition of an aldosterone antagonist) (Table 1).

**Table 1:** Patient demographic and clinical characteristics at baseline

Characteristic	Patients (n = 425)
Age, years	63.0 ± 10.6
Male sex, n (%)	309 (72.7)
Non-ischaemic heart failure aetiology, n (%)	258 (60.7)
LVEF, %	26.2 ± 6.1
LVEDD, mm	70.3 ± 10.0
Left ventricular dyssynchrony, n (%)	
Total	307 (72.2)
Persistent after CRT	51 (16.6)
Atrial fibrillation, n (%)	83 (19.5)
Baseline heart rate (/minute)	75.3 ± 14.4
Baseline QRS-duration (milliseconds)	167.8 ± 21.9
Heart failure medication, n (%)	
β-blocker	405 (95.3)
ARB or ACE inhibitor	423 (99.5)
Aldosterone antagonist	332 (78.1)

Values are mean ± standard deviation, or number of patients (%)

*ACE* angiotensin-converting enzyme, *ARB* angiotensin receptor blocker, *CRT* cardiac resynchronisation therapy, *LVEDD* left ventricular end-diastolic pressure, *LVEF* left ventricular ejection fraction

### Mechanical dyssynchrony at baseline

Left ventricular dyssynchrony (AR or SF at baseline echocardiography) was present in 307 patients (72.2%) (Table 1). Of those with mechanical dyssynchrony, 202 (65.8%) with were male. However, the prevalence of mechanical dyssynchrony was higher in females than in males (105/116 [90.5%] vs. 201/309 [65.4%]; p < 0.001). In addition, the prevalence of mechanical dyssynchrony was highest in the youngest patients, being 95% in those aged ≤ 40 years, 73% in those aged 41–50 years, 77% in those aged 51–60 years, 70% in those aged 61–70 years, and 67% in those aged ≥ 70 years (p = 0.08).

### Mechanical dyssynchrony after CRT implantation

At the 12-month follow-up, there was no longer any mechanical dyssynchrony on echocardiography in 256 of the 307 patients (83.4%) who showed this prior to CRT implantation, and mechanical dyssynchrony persisted in 51/307 patients (16.6%) patients despite optimal applied CRT adaptation. The proportion of patients in whom mechanical dyssynchrony resolved was similar for males versus females (83% vs. 84%; p = 0.88), but higher in older versus younger patients (dyssynchrony resolved in 58%, 74%, 83%, 87% and 88% of patients aged ≤ 40, 41–50, 51–60, 61–70 and > 70 years, respectively; p = 0.015).

### Events and survival

Five patients (1.2%) underwent LVAD implantation, 13 (3.1%) underwent heart transplantation and one (0.2%) had device explantation. Mean overall survival was 95.9 ± 52.9 months, and was significantly longer in women than in men (109.1 ± 52.4 vs. 90.9 ± 52.4 months, p = 0.002) and in patients aged ≤ 60 versus > 60 years (110.6 ± 53.7 vs. 88.6 ± 51.1 months; p < 0.001).

Mean survival was significantly longer in patients with versus without mechanical dyssynchrony at baseline (106.2 ± 52.0 vs. 68.9 ± 45.4 months (p < 0.001; Fig. 2). Patients for whom mechanical dyssynchrony resolved after CRT had the longest mean overall survival of any group, at 111.6 ± 51.2 months. In contrast, patients with persisting mechanical dyssynchrony despite CRT had the shortest mean survival duration (79.7 ± 47.6 months; p < 0.001 vs. patients for whom mechanical dyssynchrony resolved) (Fig. 3).

### Multivariable analysis

Multivariable analysis included all echocardiographic parameters, plus clinical parameters including age, heart failure aetiology, NYHA functional class, baseline LVEF, follow-up duration, and evidence of mechanical dyssynchrony as independent variables. In the first model, which included all variables, age, evidence of mechanical dyssynchrony and heart failure aetiology were found statistically significant independent predictors of survival (F-ratio 13.225, p < 0.001) (Table 2). In addition, follow-up time was a marginally significant variable. Model two included variables that were statistically significant in the first model, and the F-distribution ratio and statistical significance levels increased (F-ratio 21.245, p < 0.001) (Table 2). Model 3 included only variables with the greatest impact (age and evidence of mechanical dyssynchrony), and these were found to be the strongest predictors of survival in patients with HFrEF after CRT implantation (F-ratio 39.34, p < 0.001) (Table 2).

**Table 2:** Multivariable analysis models for the association between various parameters and overall survival

Variables	Regression coefficient	t	p value
Model 1			
(Constant)	144.28	5.323	< 0.001
Age	–1.003	–4.075	< 0.001
NHYA class	–9.586	–1.563	0.119
Heart failure aetiology	–14.211	–2.515	0.012
Baseline LVEF	0.68	1.545	0.123
Follow-up time	0.622	1.717	0.087
Mechanical dyssynchrony	32.512	5.206	< 0.001
Model 2			
(Constant)	140.799	8.905	< 0.001
Age	–1.087	–4.737	< 0.001
Heart failure aetiology	–9.772	–1.933	0.054
Follow-up time	0.42	1.418	0.157
Mechanical dyssynchrony	31.328	5.791	< 0.001
Model 3			
(Constant)	147.396	9.624	< 0.001
Age	–1.203	–5.358	< 0.001
Mechanical dyssynchrony	33.654	6.322	< 0.001

*LVEF* left ventricular ejection fraction, *NYHA* New York Heart Association

## Discussion

This is the first trial to investigate the effect of mechanical dyssynchrony (based on the presence of AR and SF on echocardiography) on the response to, and survival after, CRT in patients with HFrEF. Along with age, mechanical dyssynchrony in echocardiography before CRT implantation was a significant predictor of long-term survival. Furthermore, patients who had mechanical dyssynchrony that resolved in the 12 months after CRT had the best long-term survival duration, which was significantly longer than that in patients with persisting mechanical dyssynchrony throughout 12-month follow-up.

CRT is a well-established therapy for patients who have advanced symptomatic HFrEF despite optimal medical therapy [14, 15]. In this setting, CRT has been shown to improve exercise capacity and quality of life [16–21], and reduce mortality [16, 19, 22–24]. However, up to 30–50% of patients do not respond and clinically benefit from CRT [1, 2] despite optimal programming and having an indication for this therapy based on guideline recommendations [25].

Response to CRT is inconsistently defined in the literature and there is a lack of clear definitions [1, 26–29]. Moreover, CRT response rates vary between indications, from 65% in patients with an indication that has a class I guideline recommendation to 38% in patients with a class IIb guideline indication [30]. The current guideline selection criteria for CRT therapy are still mainly based on QRS duration from a resting electrocardiogram (ECG), especially considering LBBB [15]. LBBB occurs in 20–30% of patients with heart failure and is associated with increased mortality [31–33]. However, it has been suggested that up to 30% of patients included in major CRT trials did not have LBBB based on a strict definition [34]. Similarly, a comparison of four different LBBB definitions from different large trials and current guidelines found that only 13% were consistent with the strict LBBB definition [35]. Thus, based on currently available data, LBBB alone is not a reliable indicator of response to CRT and consideration of additional parameters is warranted to increase CRT response rates in patients with heart failure [28, 29, 36].

Since early in the development of CRT there have been various attempts to expand the use of echocardiography-based assessment to better identify individuals likely to response to CRT, and to include parameters of mechanical dyssynchrony. However, these parameters have not been fully studied and is probably why evaluation of mechanical dyssynchrony is not included in current guideline recommendations [14]. The PROSPECT trial raised concerns about the usefulness of single measures of echocardiography-derived mechanical dyssynchrony to improve patient selection for CRT [4], and the large ECHO-CRT trial reported adverse outcomes in patients with a narrow QRS who underwent CRT but there was no clear result with respect to mechanical dyssynchrony [5]. However, both AR and SF as indicators of mechanical dyssynchrony that have been shown to have predictive value over short- and medium-term follow-up after CR [13], but there was not previously any data including long-term follow-up and assessment of mortality. Both of these novel echocardiographic parameters are routinely qualitatively recognisable and easily quantitatively measurable in daily practice because they directly reflect the mechanical impact of LBBB [9, 11]. Many other proposed echocardiography parameters are complex to obtain or time-consuming to analyse, whereas mechanical dyssynchrony can be detected in regular 2D mode and in M-mode or speckle tracking [10, 13].

The close connection between mechanical dyssynchrony and underlying LBBB suggests its predictive potential, but no trial has yet determined the impact of mechanical dyssynchrony on mortality. In addition to reporting on echocardiography changes related to mechanical dyssynchrony after 12 months, this study also includes a 10-year evaluation of overall mortality. The presence of mechanical dyssynchrony, including AR and/or SF on the baseline echocardiogram, was the most robust predictor of response to CRT and long-term survival after CRT in patients with HFrEF. We also report for the first time that patients who had correction of mechanical dyssynchrony at 12 months after CRT implantation had the best long-term survival rate. Other findings from the current study are consistent with various previous studies, including better outcomes in women versus men and in those with non-ischaemic versus ischaemic HF aetiology [28, 37, 38, 39].﻿

Unexpectedly, we found younger patients to have a higher persistence of mechanical dyssynchrony despite optimal CRT therapy, which was associated with worse prognosis in this cohort. Comparable data are not currently available in this field, which is why we believe that large and multicenter trials are needed to study this topic in more detail. Mechanical dyssynchrony could be a new promising parameter that may help to improve CRT response in HF patients and in particular to improve survival in HF CRT patients.

### Limitations

This prospective study had a single-centre, observational, nonrandomised design. Echocardiography was performed before and after CRT implantation but did not influence the CRT indication or programming in a systematic way. In addition, other important reasons for non-response to CRT such as lead position or rate of biventricular pacing were not systematically followed in our study, and atrioventricular-optimisation was not routinely performed. Furthermore, the individual scar load in patients with ischaemic HF was not quantified. Finally, there was no distinction made between cardiac and non-cardiac causes of death and comorbidities have not been collected throughout in this patient population. Anti-arrhythmic medication has not been collected throughout.

## Conclusion

Novel echocardiography parameters indicating mechanical dyssynchrony documented in CRT recipients predicted subsequent long-term survival in patients with HFrEF after CRT implantation. In addition, correction of mechanical dyssynchrony in the first 12 months after CRT is essential for good long-term survival. These findings support the undertaking of a prospective, randomised, controlled, multicentre trial to better evaluate the clinical implications of determining mechanical dyssynchrony prior to CRT patients with HFrEF.
